# Burden and seasonality of primary and secondary symptomatic common cold coronavirus infections in Nicaraguan children

**DOI:** 10.1111/irv.13086

**Published:** 2022-12-09

**Authors:** Aaron M. Frutos, Angel Balmaseda, Nivea Vydiswaran, Mayuri Patel, Sergio Ojeda, Andrew Brouwer, Rebecca Tutino, Shuwei Cai, Kevin Bakker, Nery Sanchez, Roger Lopez, Guillermina Kuan, Aubree Gordon

**Affiliations:** ^1^ Department of Epidemiology, School of Public Health University of Michigan Ann Arbor Michigan USA; ^2^ Health Center Sócrates Flores Vivas Ministry of Health Managua Nicaragua; ^3^ Laboratorio Nacional de Virología, Centro Nacional de Diagnósticoy Referencia Ministry of Health Managua Nicaragua; ^4^ Sustainable Sciences Institute Managua Nicaragua

**Keywords:** child health, cohort study, coronavirus, global health, infant health, Latin America

## Abstract

**Background:**

The current SARS‐CoV‐2 pandemic highlights the need for an increased understanding of coronavirus epidemiology. In a pediatric cohort in Nicaragua, we evaluate the seasonality and burden of common cold coronavirus (ccCoV) infection and evaluate likelihood of symptoms in reinfections.

**Methods:**

Children presenting with symptoms of respiratory illness were tested for each of the four ccCoVs (NL63, 229E, OC43, and HKU1). Annual blood samples collected before ccCoV infection were tested for antibodies against each ccCoV. Seasonality was evaluated using wavelet and generalized additive model (GAM) analyses, and age–period effects were investigated using a Poisson model. We also evaluate the risk of symptom presentation between primary and secondary infections.

**Results:**

In our cohort of 2576 children from 2011 to 2016, we observed 595 ccCoV infections and 107 cases of ccCoV‐associated lower respiratory infection (LRI). The overall incidence rate was 61.1 per 1000 person years (95% confidence interval (CI): 56.3, 66.2). Children under two had the highest incidence of ccCoV infections and associated LRI. ccCoV incidence rapidly decreases until about age 6. Each ccCoV circulated throughout the year and demonstrated annual periodicity. Peaks of NL63 typically occurred 3 months before 229E peaks and 6 months after OC43 peaks. Approximately 69% of symptomatic ccCoV infections were secondary infections. There was slightly lower risk (rate ratio (RR): 0.90, 95% CI: 0.83, 0.97) of LRI between secondary and primary ccCoV infections among participants under the age of 5.

**Conclusions:**

ccCoV spreads annually among children with the greatest burden among ages 0–1. Reinfection is common; prior infection is associated with slight protection against LRI among the youngest children.

## 1 INTRODUCTION

The SARS‐CoV‐2 pandemic underscores the need for understanding human coronavirus epidemiology. The four common cold coronaviruses (ccCoVs), NL63, 229E, OC43, and HKU1, are generally associated with upper respiratory tract infections^1^, ^2^, ^3^, ^4^ but have also been associated more severe lower respiratory tract infections (LRI).^5^, ^6^, ^7^, ^8^ Following the detection of NL63 and HKU1 in the 2000s, OC43 is the most frequently detected globally, whereas 229E is the least and is primarily detected among individuals with severe infections or weakened immune systems.^4^, ^7^, ^8^, ^9^, ^10^, ^11^, ^12^, ^13^, ^14^, ^15^, ^16^ ccCoVs are split into two, genetically similar groups, alpha (NL63 and 229E) and beta (OC43 and HKU1)^2^; prior work has identified cross‐reactive antibodies within and between groups.^17^ However, it is unclear whether immunity to one of the ccCoV, whether within alpha and beta groups or across, protects against infection with another.

Younger children have higher rates of symptomatic and severe illness associated with ccCoV infection compared with older children and adults.^7^, ^10^, ^18^ By age 3, most children have had their first ccCoV infection, and by age 6, children typically have antibodies against each of the four ccCoV types.^19^, ^20^ ccCoV infections occur repeatedly throughout life, suggesting the lack of long‐lasting sterilizing immunity produced by natural infection.^21^ Declining antibody levels following primary ccCoV infection may explain frequent ccCoV reinfection in children.^20^ The clinical significance of primary versus secondary ccCoV infections in children is not well understood.

Many large ccCoV studies lack a well‐defined study population and rely on reporting from hospitals, healthcare systems, and passive surveillance networks; these studies detect and report on the epidemiology of more severe ccCoV infections.^3^, ^6^, ^7^, ^8^, ^9^, ^11^, ^13^, ^14^ Studies conducted in temperate locations report consistent annual seasonal peaks during winter months, similar to other common respiratory pathogens; ccCoV spread in other climates, however, does not appear to follow similar patterns and drivers of ccCoV seasonality remain unknown.^3^, ^4^, ^7^, ^8^, ^9^, ^10^, ^11^, ^22^

Here, we describe the incidence and seasonality of symptomatic ccCoV infections and evaluate risk of symptom presentation of between primary and secondary ccCoV infections in a community‐based pediatric cohort in Managua, Nicaragua, from 2011 to 2016.

## 2 METHODS

The Nicaraguan Pediatric Influenza Cohort (NPICS) is an ongoing prospective cohort study of children aged 0–14 years in Managua, Nicaragua, which has a tropical, urban environment. Previous work has detailed descriptions of study protocols.^23^ Briefly, children aged 0–12 were enrolled in 2011, and newborns are enrolled monthly. Parents agreed to bring enrolled children to the study health center, Health Center Sócrates Flores Vivas, at the first signs of a fever. Children age out of the cohort on their 15th birthday. This analysis uses data collected January 1, 2011 to December 31, 2016.

Study personnel collected nasal and oropharyngeal swabs (oropharyngeal only if under 6 months) from participants if they met any one of the testing criteria: (1) fever (temperature of 37.8°C or greater) or feverishness and cough, sore throat, and/or rhinorrhea; (2) fever or feverishness and under 2 years old; (3) severe respiratory symptoms (i.e., pneumonia, chest indrawing, wheezing, and apnea) evaluated by a study physician; and (4) hospitalization with respiratory symptoms or sepsis. Laboratory personnel at the University of Michigan tested samples using reverse transcriptase‐polymerase chain reaction (PCR) for the four seasonal ccCoVs following the Centers for Disease Control and Prevention protocol.^24^ Respiratory symptoms are recorded from each clinic visit by study physicians as well as from symptom diaries by parents/guardians. Participants with diagnosed cases of pneumonia, bronchiolitis, bronchitis, or bronchial hyperreactivity were considered to have LRI.^25^

Blood samples were collected annually from participants between February and April each year. To evaluate the frequency of secondary ccCoV infections, blood samples that were collected within 1 year before a ccCoV PCR+ infection were tested for IgG antibodies to the spike protein for each ccCoV via an enzyme‐linked immunosorbent assay following previously developed protocols.^26^ Results from blood samples may be paired with multiple PCR+ infections if a participant had multiple PCR+ infections within a year.

Person time was calculated as the number days between the participants' enrollment and exit from the study. Exit dates were determined as participants' 15th birthday for NPICS, the day the participant withdrew from the study, or was lost to follow‐up. In cases of loss‐to‐follow‐up, the midpoint between the date of last contact with the participant and the start of the annual survey collection as the exit date was used. Participants did not contribute person time for 28 days following a PCR positive sample. Person time was calculated for all ccCoV infections and separately for each coronavirus. To identify significant seasonal patterns, wavelet analyses with pink noise and a log +1 transformation were used. To examine if the seasonality of one ccCoV impacted the seasonality of another, a cross‐wavelet analysis was conducted. Using time‐series data, wavelets can be used to identify periodic signals; cross‐wavelet analysis allows us to evaluate the temporal relationship between two time series.^27^, ^28^, ^29^ A generalized additive model was used to identify peak months for each group and type. To calculate incidence rates, a Poisson model was used. Crude rates and rates adjusted for period and for age were calculated; age was adjusted for using B‐splines; age period provided better model fit than age cohort or period cohort. Crude and fitted incidence rates were displayed using hexamaps to visualize age–period‐cohort (APC) trends.^30^

To evaluate differences in symptom presentation between primary and secondary infections, symptom presentation risk was compared among those with blood samples collected within a year before a ccCoV PCR+ infection. Secondary infections were defined as PCR+ infections following a previous PCR+ infection with the same ccCoV type or presence of type‐specific IgG spike antibodies before infection. PCR+ infections in children under the age of 1 without a collected blood before infection were considered primary infections. Risk ratios were calculated from a generalized estimating equation log‐binomial model adjusted for age linearly; this model was restricted to participants under the age of 5.

We used R version 4.1.1 to create figures as well as conduct the wavelet, GAM, and incidence analyses. All other analyses occurred in SAS version 9.4 (SAS Institute Inc.).

## 3 RESULTS

From 2011 to 2016, there were 2576 NPICS participants who contributed 7309 person years. On average, about 3% of participants withdrew from the study or were lost to follow‐up per year (range 2–6%), and over the 6 years, there were six deaths (Figure S1). Approximately 50% of participants were female. There were between 1436 and 1776 active participants each month. (Figure S2).

Study personnel collected 9018 respiratory samples of which 8803 (97.6%) had sufficient sample remaining to test all four ccCoVs. Six hundred ten (6.8%) were positive for ccCoVs. We detected 595 distinct coronavirus infections and 28 ccCoV coinfections (sample positive for two or more ccCoVs) among 476 participants. OC43 was the most common ccCoV detected (*n* = 323; 52.9%) and followed by NL63 (*n* = 163; 26.7%), 229E (*n* = 86; 14.1%), and HKU1 (*n* = 69; 11.3%) (Table 1). There were 107 cases of ccCoV‐associated LRI and 23 hospitalizations.

**TABLE 1:** Study participants and samples collected by year

	2011	2012	2013	2014	2015	2016	Total
**Participants**	1579	1653	1790	1950	1895	1874	2576
Female (%)	795 (50.3)	830 (50.2)	903 (50.5)	973 (49.9)	946 (49.9)	944 (50.3)	1307 (50.7)
**Person years**	1480	1531	1599	1684	1676	1717	9687
Age 0	93	63	117	123	112	109	616
Age 1	127	109	57	109	119	122	642
Ages 2–4	330	319	349	325	353	361	2037
Ages 5–9	597	576	525	551	559	597	3404
Ages 10–14	345	464	551	577	533	528	2998
**Respiratory samples**	1423	1448	1583	1650	1566	1348	9018
ccCoV PCR+ (%)	94 (6.6)	111 (7.7)	113 (7.1)	100 (6.1)	109 (7.0)	83 (6.2)	610 (6.8)
NL63 (%^a^)	15 (16.0)	29 (26.1)	43 (38.5)	34 (34.0)	24 (22.0)	18 (21.7)	163 (26.7)
229E (%^a^)	19 (20.2)	13 (17.4)	13 (11.5)	7 (7.0)	9 (8.3)	15 (18.1)	86 (14.1)
OC43 (%^a^)	54 (57.4)	74 (66.7)	37 (32.7)	58 (58.0)	54 (49.5)	46 (55.4)	323 (52.9)
HKU1 (%^a^)	7 (4.3)	1 (0.9)	22 (19.5)	6 (6.0)	26 (23.9)	7 (8.4)	69 (11.3)

Abbreviations: ccCoV, common cold coronavirus; PCR, polymerase chain reaction.

^a^ Does not sum to 100 because of codetections.

**FIGURE 1:**
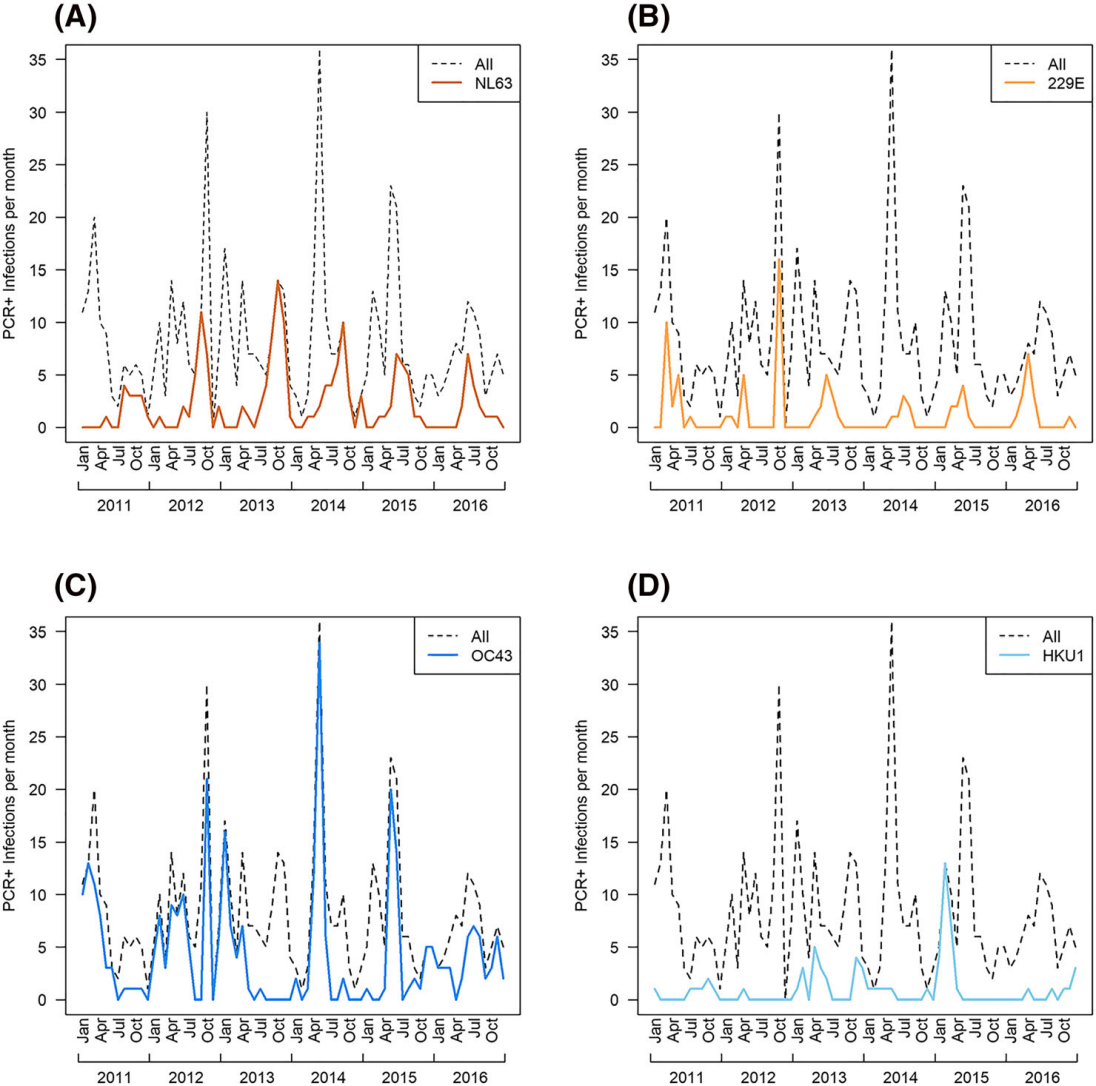
Monthly common cold coronavirus (ccCoV) PCR+ infections by type, 2011–2016. Symptomatic ccCoV infections over time. Dashed line represents monthly sum of all ccCoV PCR+ infections during the study period. (A) NL63 PCR+ infections; (B) 229E PCR+ infections; (C) OC43 PCR+ infections; and (D) HKU1 PCR+ infections

**FIGURE 2:**
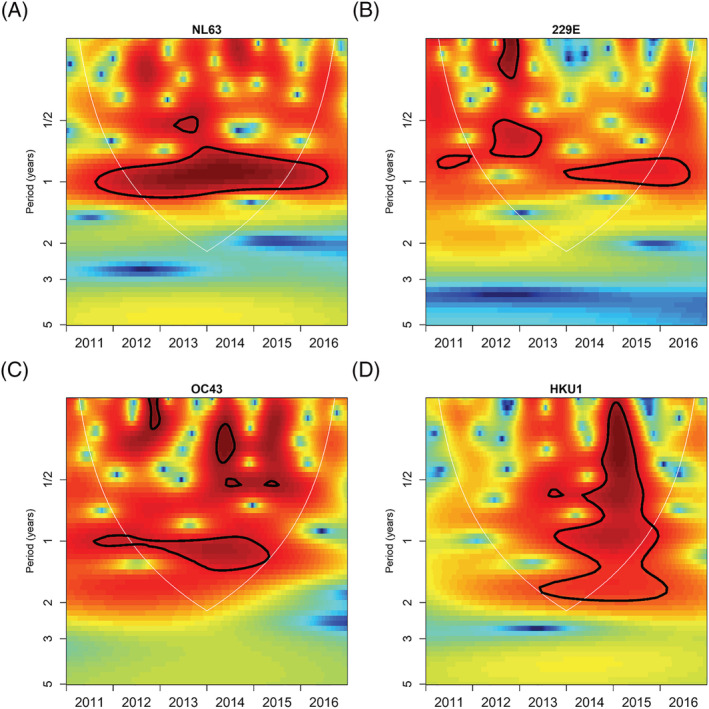
Wavelet analysis by common cold coronavirus type. Wavelet analysis conducted separately by common cold coronavirus type to identify type‐specific periodicity. Red represents dominant periods, and the area circled in black lines represent significant periodicity. Only the area within the light gray semicircle (the wavelet cone of influence) can be interpreted. (A) NL63 wavelet analysis; (B) 229E wavelet analysis; (C) OC43 wavelet analysis; and (D) HKU1 wavelet analysis

There was no clear season to ccCoV circulation, with cases presenting in every month of the study period (Figure 1). NL63, 229E, and OC43 circulated annually throughout the study period. NL63 generally peaked in the last 6 months of the year, but there was no identified general peak month for the other ccCoVs (Figures 2 and S4). Cross‐wavelet analysis indicated that 229E peaks generally occur 3 months before NL63; we also found that NL63 and OC43 peaks occurred approximately 6 months apart from 2011 to 2013 but shifted to 3 months apart from 2014 to 2015 (Figure S5).

**FIGURE 3:**
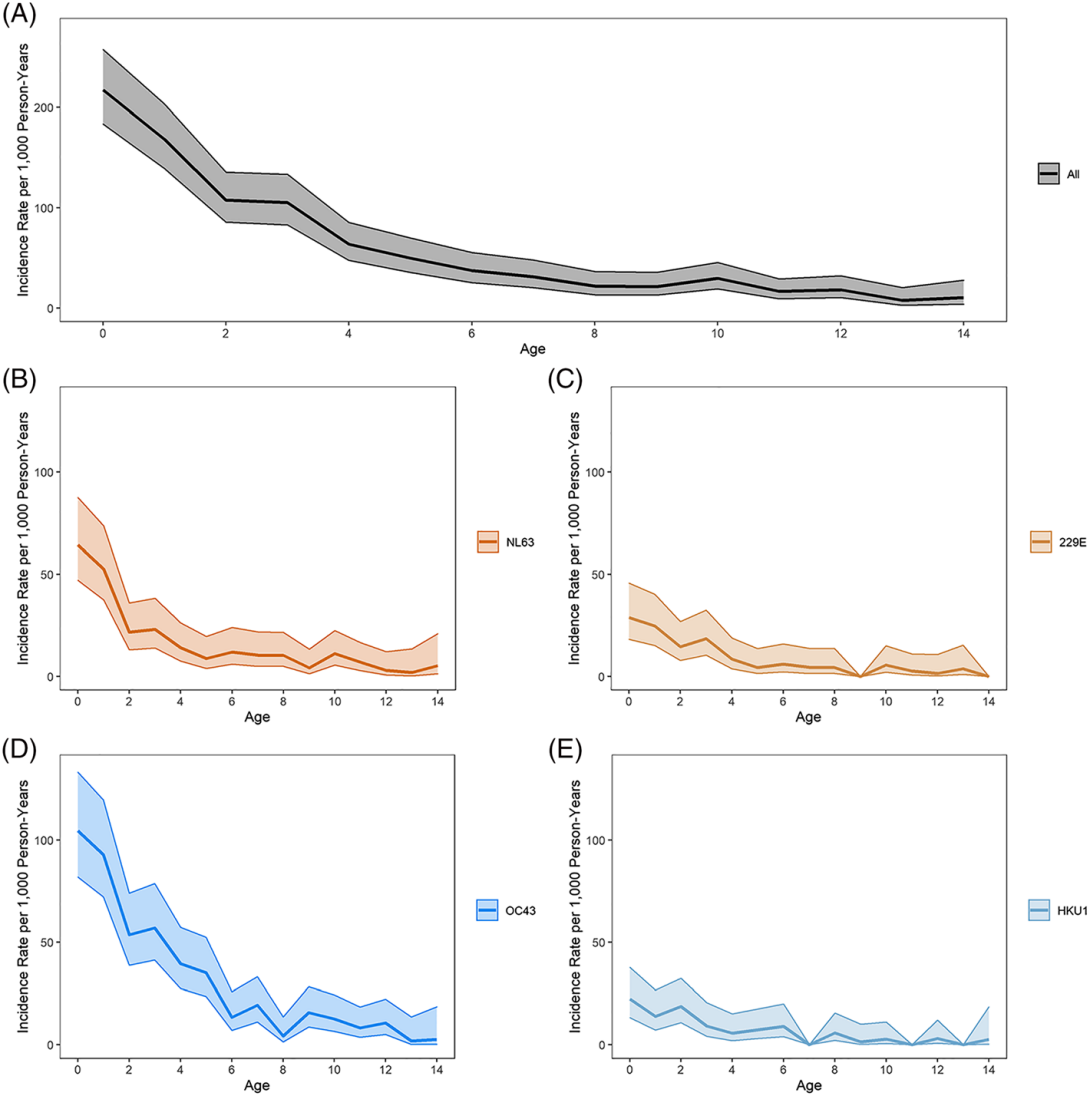
Common cold coronavirus (ccCoV) incidence rates by age and type. Incidence rates (per 1000 person years) of PCR+ ccCoV infections for all ccCoV infections and by type using 1‐year age groups. Shaded area represents 95% confidence intervals. (A) All ccCoV; (B) NL63; (C) 229E; (D) OC43; and (E) HKU1

**FIGURE 4:**
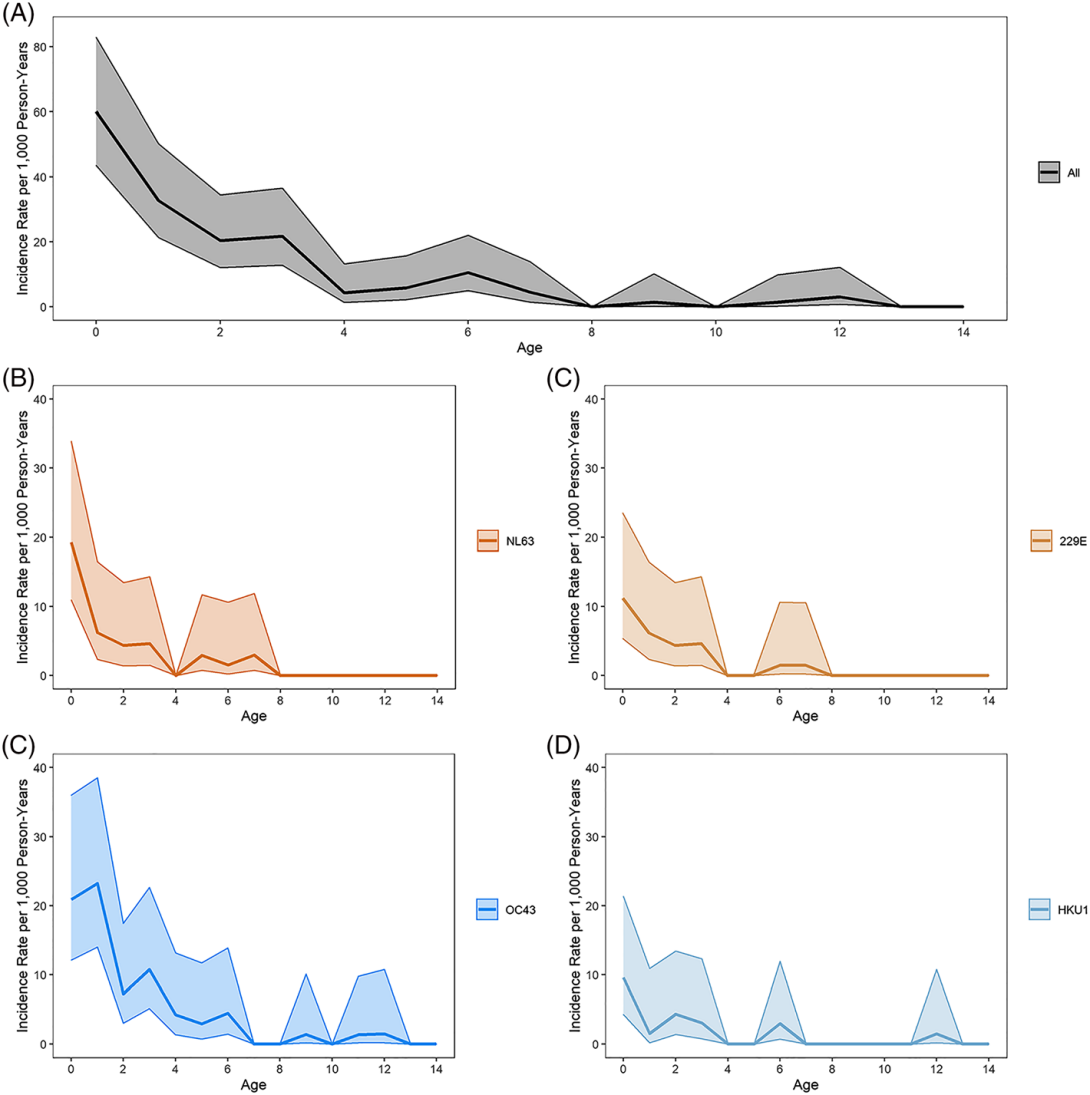
Common cold coronavirus (ccCoV)‐associated lower respiratory infection incidence rates by age and type. Incidence rates (per 1000 person years) of PCR+ ccCoV‐associated lower respiratory infections (LRI) for all ccCoV infections and by type using 1‐year age groups. Shaded area represents 95% confidence intervals. (A) All ccCoV‐associated LRI; (B) NL63‐associated LRI; (C) 229E‐associated LRI; (D) OC43‐associated LRI; and (E) HKU1‐associated LRI

Overall incidence of symptomatic ccCoV infection was 61.1 per 1000 person years (95% CI: 56.3, 66.2). Incidence was the highest among the youngest participants and sharply decreased with increasing age. The incidence rate among those aged 4 (63.9, 95% CI: 47.7, 85.5) was less than a third of the rate among those less than 1 year old (217.4, 95% CI: 183.6, 257.6), and the rate among those aged 8 (22.0.3, 95% CI: 13.3, 36.5) is about a third of the rate among those aged 4. This age pattern is similar for each ccCoV type (Figure 3). Incidence rates between males and females were similar (Table S1). ccCoV‐associated LRI incidence was 8.9 per 1000 person years (95% CI: 7.2, 11.0). LRI incidence was also the highest among the youngest participants, with all ccCoV‐associated LRI incidence following a similar age pattern as symptomatic infection incidence (Figure 4). There was also no difference in ccCoV‐associated LRI incidence by sex (Table S1).

**FIGURE 5:**
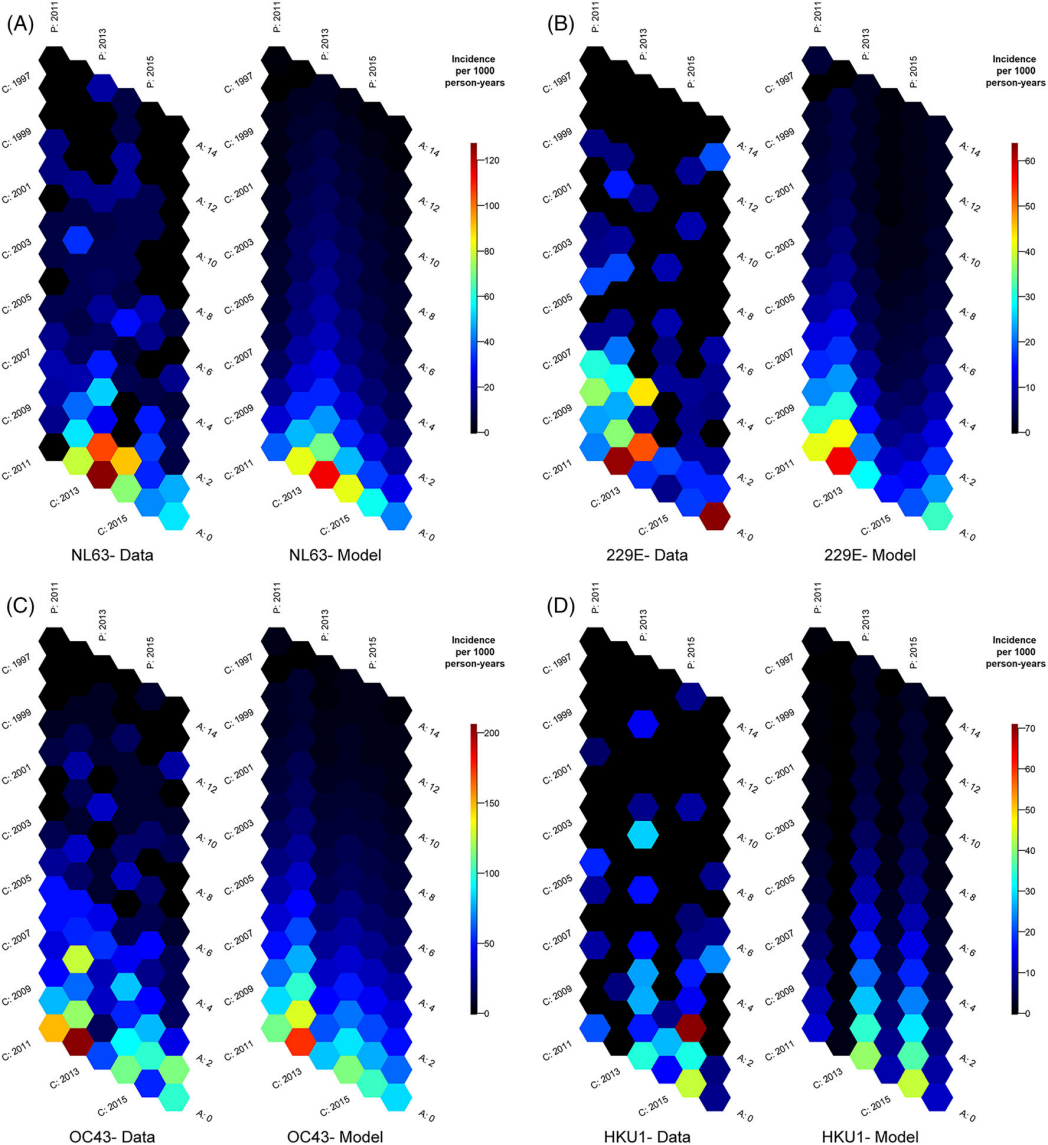
Common cold coronavirus (ccCoV) incidence rate hexamaps. Visualizing ccCoV incidence rates (per 1000 person years) by type as a function of age, calendar year, and birth year. Hexamaps with incidence rates from raw data are presented on the left within each ccCoV type's panel. Predicted incidence rates from age–period model are presented on the right within each ccCoV type's panel. (A) NL63; (B) 229E; (C) OC43; and (D) HKU1

Although it has been shown that people are repeatedly infected with ccCoVs, we hypothesized that the breadth of immunity would increase as children accumulate exposures to the same type, resulting in a decrease in the incidence of cases. Age–period‐cohort analysis suggests that incidence declines sharply until around age 6 when incidence rates decline more slowly (Figures 5 and S6). At age 6, ccCoV incidence is less than 30% of infant ccCoV incidence. Symptomatic infections are relatively uncommon in individuals older than 10 years old. Periods that had higher incidence for a specific ccCoV (e.g., NL63 in 2013 and HKU1 in 2015) had higher incidence among all participants, including older children (Figure S7). Compared with others, cohorts with lower incidence at under 1 year of age old (e.g., NL63 and 2011 cohort and OC43 and 2013 cohort) had generally higher incidence at age 1.

To determine whether an infection was a primary or secondary ccCoV infection, we tested 406 blood samples that were collected within 1 year before 434/595 (72.9%) ccCoV PCR+ infections. Of the 161 PCR+ infections without a blood sample collected within 1 year, 108 (67%) were among participants less than 1 year old. Overall, most children experiencing an infection had at least one prior ccCoV infection. OC43 antibodies were detected most frequently (*n* = 316, 77.8%) and then NL63 (*n* = 286, 70.4%), HKU1 (*n* = 278, 68.5%), and 229E (*n* = 257, 63.3%). Three hundred infections (69.1%) occurred in participants who had preexisting antibodies against the infecting ccCoV type, ranging from 73.5% of OC43 infected participants to 63.1% of 229E acute infections (Table 2). Seropositivity was over 50% for each ccCoV for those aged 2 and older (Figure S3).

**TABLE 2:** ccCoV antibodies before PCR+ infection by year

	2011	2012	2013	2014	2015	2016	Total
**Blood samples before ccCoV PCR+**	43	84	75	71	78	55	406
NL63 antibodies (%)	29 (67.4)	57 (67.9)	57 (76.0)	55 (77.5)	54 (69.2)	34 (61.8)	286 (70.4)
229E antibodies (%)	28 (65.1)	58 (69.1)	53 (70.7)	43 (60.6)	42 (53.9)	33 (66.0)	257 (63.3)
OC43 antibodies (%)	34 (79.1)	69 (82.1)	67 (89.3)	48 (67.6)	55 (70.5)	43 (78.2)	316 (77.8)
HKU1 antibodies (%)	32 (74.4)	68 (81.0)	57 (76.0)	41 (57.8)	52 (66.7)	28 (50.9)	278 (68.5)
**PCR+ infections**	44	92	79	79	81	59	434
Antibodies against ccCoV type (%)	30 (68.2)	70 (76.1)	61 (77.2)	49 (62.0)	54 (66.7)	36 (61.0)	300 (69.1)
**NL63**	13	21	27	25	18	13	117
NL63 antibodies (%)	10 (76.9)	14 (66.7)	17 (63.0)	17 (68.0)	15 (83.3)	6 (46.2)	79 (67.5)
**229E**	13	19	10	6	7	10	65
229E antibodies (%)	8 (61.5)	14 (73.7)	7 (70.0)	4 (66.7)	2 (28.6)	6 (60.0)	41 (63.1)
**OC43**	13	62	25	48	46	32	226
OC43 antibodies (%)	7 (53.9)	51 (82.3)	24 (96.0)	30 (62.5)	32 (69.6)	22 (68.8)	166 (73.5)
**HKU1**	5	1	18	5	14	7	50
HKU1 antibodies (%)	5 (100)	1 (100)	14 (77.8)	1 (20.0)	7 (50.0)	5 (71.4)	33 (66.0)

Abbreviations: ccCoV, common cold coronavirus; PCR, polymerase chain reaction.

By age 5, 96.7% of symptomatic infections were type‐specific secondary infections (range 95.0% to 100.0% by type). Indeed, for the most common types, OC43 and NL63, 54.6% and 67.7%, of symptomatic infections are secondary infections by age 2; for 229E and HKU1, only 14.3% and 36.4% are secondary infections by age 2. We then examined the severity of primary versus secondary ccCoV infections, adjusted for age. Because almost all ccCoV symptomatic infections were secondary infections by age 5, we limited this analysis to participants under the age of 5. The risk of ccCoV‐associated LRI was lower among secondary infections compared with primary infections (RR: 0.90, 95% CI: 0.83, 0.97). We found that the risk of cough (RR: 1.12, 95% CI: 1.04, 1.20) and rhinorrhea (RR: 1.13, 95% CI: 1.06, 1.2) were slightly higher among those with secondary infections compared with primary infections. We found no difference in risk of measured fever, congestion, or hospitalization by serostatus prior to infection (Table 3).

**TABLE 3:** Risk of symptom presentation, secondary versus primary common cold coronavirus type infections

Symptom	Risk ratio (95% CI)
Measured fever	0.91 (0.70, 1.18)
Cough	1.12 (1.04, 1.20)
Rhinorrhea	1.13 (1.06, 1.21)
Congestion	1.12 (0.85, 1.47)
Hospitalization	0.92 (0.18, 4.64)
Lower respiratory infection	0.90 (0.83, 0.97)

## 4 DISCUSSION

This study investigates the burden and seasonality of symptomatic ccCoV infections in a community‐based pediatric cohort. This study is the longest running pediatric cohort in Central or South America that has evaluated the burden of ccCoV infections. Like other studies in non‐temperate locations, we observed ccCoV infections throughout the year,^22^, ^31^, ^32^, ^33^, ^34^ with alternating spread of different ccCoV types. Although there was no distinct ccCoV season in Managua, Nicaragua, each ccCoV type exhibited annual periodicity. We found that the two alpha coronaviruses, 229E and NL63, peaks generally do not occur at the same time. Other studies in temperate locations found that although NL63 and 229E did spread at the same time, years with a high prevalence of 229E coincided with low levels of NL63.^9^, ^35^ These results may indicate the presence of short‐term, subgroup specific cross‐reactive immunity.^36^, ^37^, ^38^, ^39^, ^40^, ^41^, ^42^

Consistent with other respiratory infections and previous research, younger children had a higher incidence of symptomatic ccCoV infections and ccCoV‐associated LRI than older children, especially within the first 2 years of life.^3^, ^7^, ^9^, ^10^, ^11^, ^12^, ^43^ We note a clear pattern of rapidly decreasing incidence of symptomatic infection until about age 6 at which point nearly all infections are secondary. Additionally, ccCoV reinfection is very common among children^20^; we found that by age 5, almost all symptomatic infections were secondary, not primary infections. Among those under 5 years of age, there was slightly lower risk of ccCoV‐associated LRI for secondary infections compared with primary infections after adjusting for age. Similarly, frequent reinfection with SARS‐CoV‐2 has also been observed among children.^44^ In a household transmission study, infection‐induced immunity was not associated with protection against SARS‐CoV‐2 infection for children.^45^ These findings suggest that although protection against ccCoV‐associated LRI develops following a primary infection, protection against symptomatic infections wanes quicker early in life^20^ but may build, lasting longer, over several exposures.

We also observed some years that had high ccCoV type‐specific incidence rates across all ages. We expect that ccCoV type‐specific genetic diversity, frequently detected among children,^46^ may explain these high incidence years. Additionally, birth cohorts that experienced lower rates of symptomatic infections for a particular ccCoV type as infants had higher rates of symptomatic illness at age 1 compared with other cohorts; this was likely a result of both annual ccCoV spread and an absence of type‐specific immunity acquired before the 1 year of age.

The main strength of this study is the size and duration of the prospective cohort. With over 9000 respiratory samples collected and over 7000 person years, we observed almost 600 ccCoV infections, exclusively among children. The 6 years of data provides sufficient power to evaluate seasonality statistically, identify annual periodicity, and evaluate the frequency of repeat ccCoV infections. The consistent cohort age structure and limited loss‐to‐follow‐up allowed us to identify age–period‐cohort trends of symptomatic ccCoV illness.

We do note some limitations in this study. Respiratory swabs were only collected when a participant presented at the clinic with symptomatic illness, thus likely missing some mild cases and underestimating the true incidence of both ccCoV infections and the frequency of reinfections in the population. However, testing participants' blood samples for ccCoV antibodies did reveal that the majority of symptomatic ccCoV infections were reinfections. Additionally, we did not examine genetic variation in ccCoVs which may help to explain seasonal variation as well as the frequency of reinfections.

Although ccCoV infections occur repeatedly throughout childhood, our understanding of coronavirus epidemiology in early life is limited. We show that ccCoV infections spread continuously throughout the year in a pediatric population in Nicaragua, with frequent reinfections; however, history of prior infection did convey protection against ccCoV‐associated LRI among those under five. Future research should focus on the early‐life development of coronavirus immunity to the contributions of viral evolution and immunity to coronavirus reinfections and immune correlates of protection against coronaviruses.

## CONFLICTS OF INTEREST

Aubree Gordon serves on an RSV vaccine scientific advisory board for Janssen Pharmaceuticals and has served on a COVID‐19 scientific advisory board for Gilead Sciences. All other authors certify no potential conflicts of interests.

## ETHICS STATEMENT

The institutional review boards at the Nicaraguan Ministry of Health and the University of Michigan approved these studies.

## AUTHOR CONTRIBUTIONS

**Aaron M Frutos:** conceptualization; formal analysis; methodology; validation; visualization. **Angel Balmaseda:** data curation; project administration; resources. **Nivea Vydiswaran:** data curation; project administration. **Mayuri Patel:** data curation; project administration. **Sergio Ojeda:** data curation; project administration. **Andrew Brouwer:** formal analysis; methodology; validation; visualization. **Rebecca Tutino:** data curation; project administration. **Shuwei Cai:** data curation; project administration. **Kevin Bakker:** formal analysis; methodology; validation; visualization. **Nery Sanchez:** data curation; project administration. **Roger Lopez:** data curation; project administration. **Guillermina Kuan:** data curation; project administration; resources. **Aubree Gordon**: conceptualization; funding acquisition; methodology; project administration; resources; supervision; validation.

## PATIENT CONSENT STATEMENT

Parents/guardians of participants provided written informed consentand children 6 years and older provided verbal assent. All consenting documents and scripts were approved by institutional review boards at the Nicaraguan Ministry of Health and the University of Michigan.

## PERMISSION TO REPRODUCE MATERIAL FROM OTHER SOURCES

This manuscript does not contain copyrighted works.

### PEER REVIEW

The peer review history for this article is available at https://publons.com/publon/10.1111/irv.13086.

## Supporting information


**Figure S1:** Participant Enter‐Exit by Year. Flow chart represent total active participants in January for each year from 2011–2016 with total number of participants entering (enrolled, re‐enrolled) and exiting (aged out, withdrawn, or deaths) the cohort.

**Figure S2:** Participation by Age, Month. Total number of monthly active participants in the cohort over the study period by age groups.

**Figure S3:** ccCoV Seropositivity by Age, Type. Proportion of participants with ccCoV‐antibodies before ccCoV PCR + infection by one year age groups and type. A: NL63, B: 229E, C: OC43, D: HKU1

**Figure S4:** Generalized Additive Model Analysis for Peak Month by ccCoV Type. Analysis uses month as the predictive variable for time series data for each ccCoV type. Dotted lines represent 95% confidence intervals. If confidence intervals at the peaks overlap with confidence intervals of the trough, there is no significant peak month. A: NL63, B: 229E, C: OC43, D: HKU1

**Figure S5:** Cross‐wavelet Analysis. Cross‐wavelet analysis of two ccCoV types to evaluate temporal relationship. White arrows pointing up at a period of 1 year represent a three‐month lag between the first list ccCoV type and the second. Arrows pointing to the left at a period of 1 year represent a six‐month lag between types. A: 229E‐NL63, B: OC43‐NL63

**Figure S6:** Age‐period Incidence Model‐ Age Effects. Predicted rate ratios by age from age‐period model by ccCoV type. Black line represents predicted rate ratios for all ccCoV infections for comparison. A: NL63, B: 229E, C: OC43, D: HKU1

**Figure S7:** Age‐period Incidence Model‐ Period Effects. Predicted rate ratios by year from age‐period model by ccCoV type. 2011 is the reference category. Black points and confidence intervals represent predicted rate ratios for all ccCoV infections for comparison.

**Table S1.** Incidence Rates by Sex

## Data Availability

Individual‐level data may be shared with outside investigators following University of Michigan IRB approval. Please contact Aubree Gordon (gordonal@umich.edu) to arrange for data access.
