# Supplementary figures and images for: Burden and seasonality of primary and secondary symptomatic common cold coronavirus infections in Nicaraguan children
Source: Influenza Other Respir Viruses. 2022 Dec 9;17(1):e13078. doi: 10.1111/irv.13086 (PMC9835451; doi:10.1111/irv.13086)

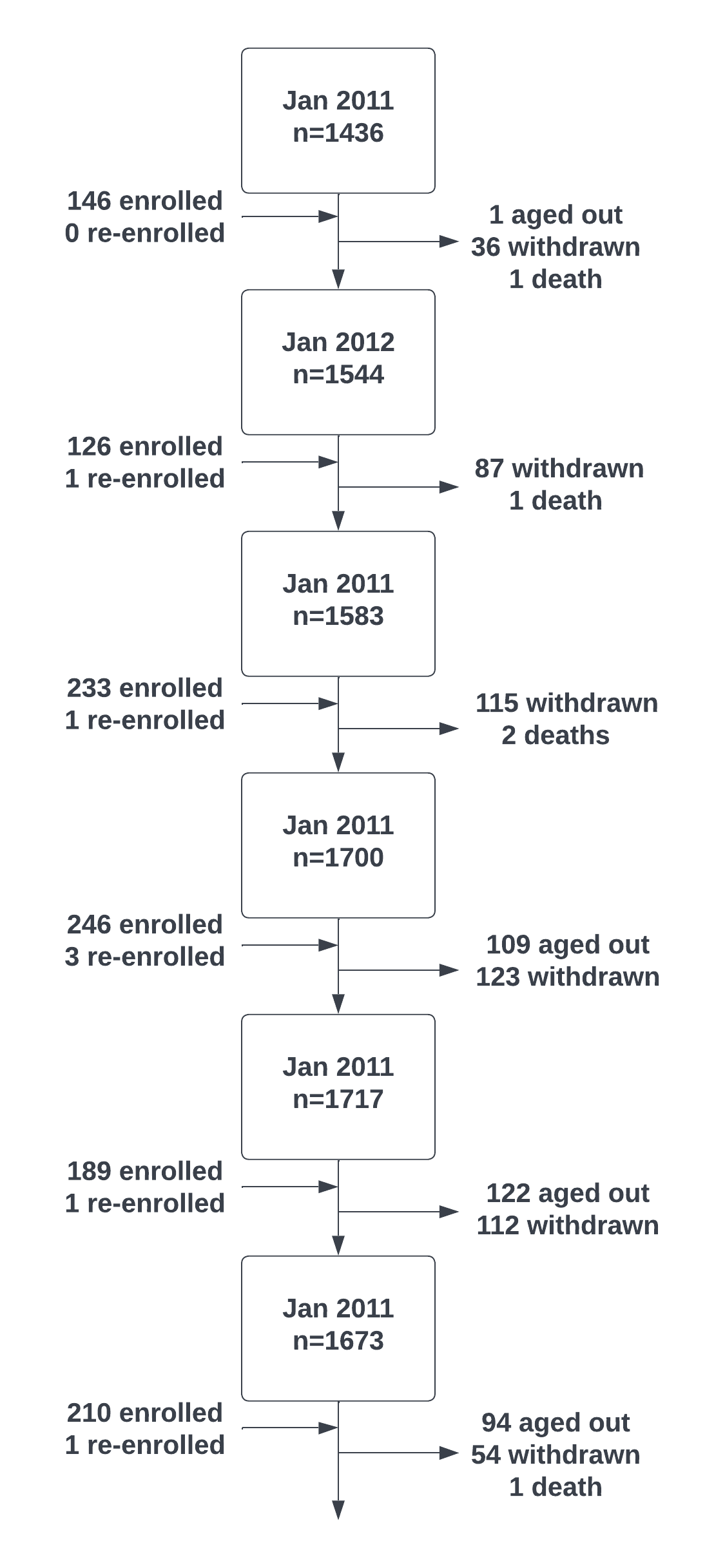

Supplement: Supplementary file 1 — Figure S1: Participant Enter‐Exit by Year. Flow chart represent total active participants in January for each year from 2011–2016 with total number of participants entering (enrolled, re‐enrolled) and exiting (aged out, withdrawn, or deaths) the cohort. [file IRV-17-0-s002.png]

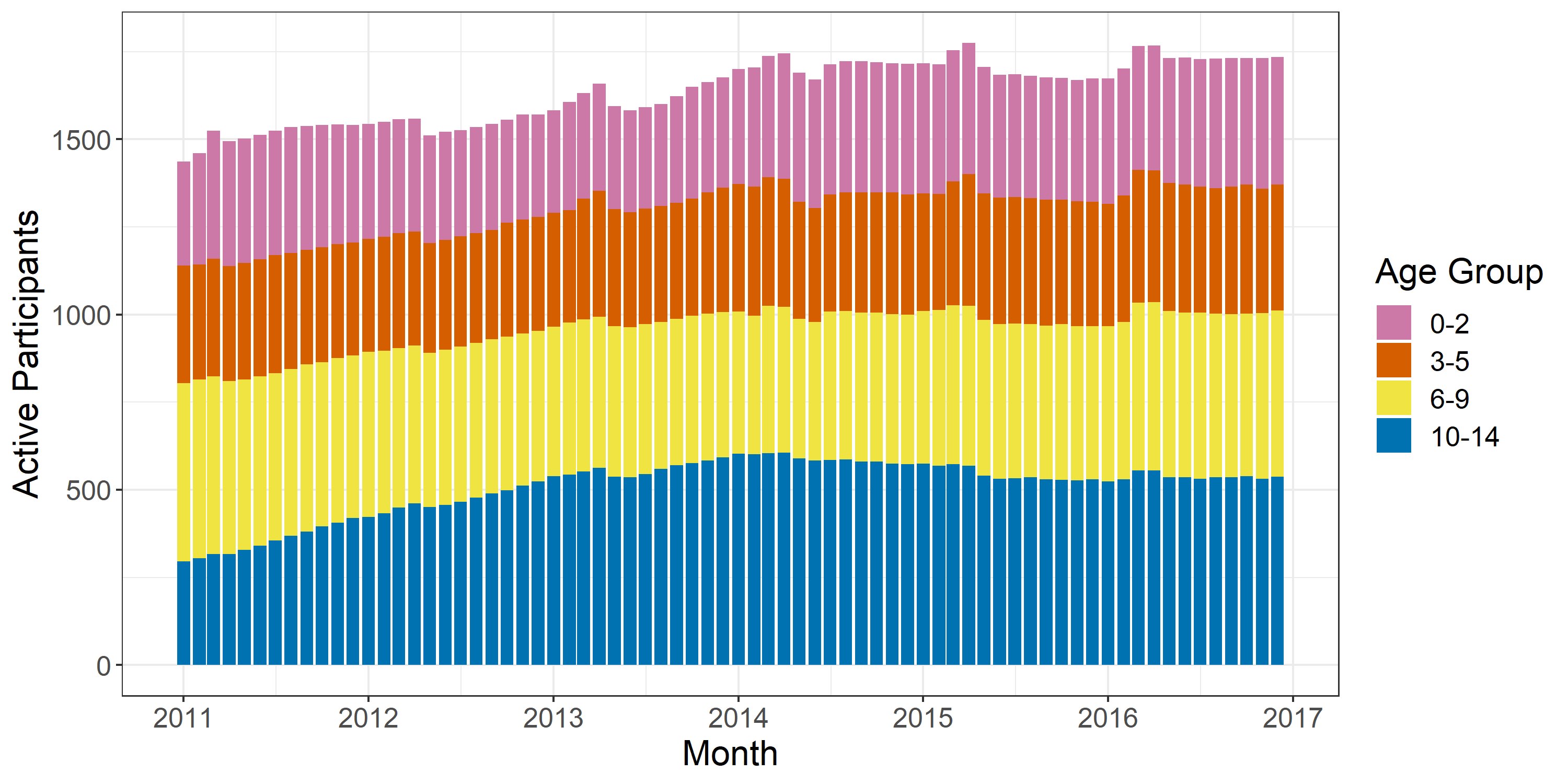

Supplement: Supplementary file 2 — Figure S2: Participation by Age, Month. Total number of monthly active participants in the cohort over the study period by age groups. [file IRV-17-0-s007.tiff]

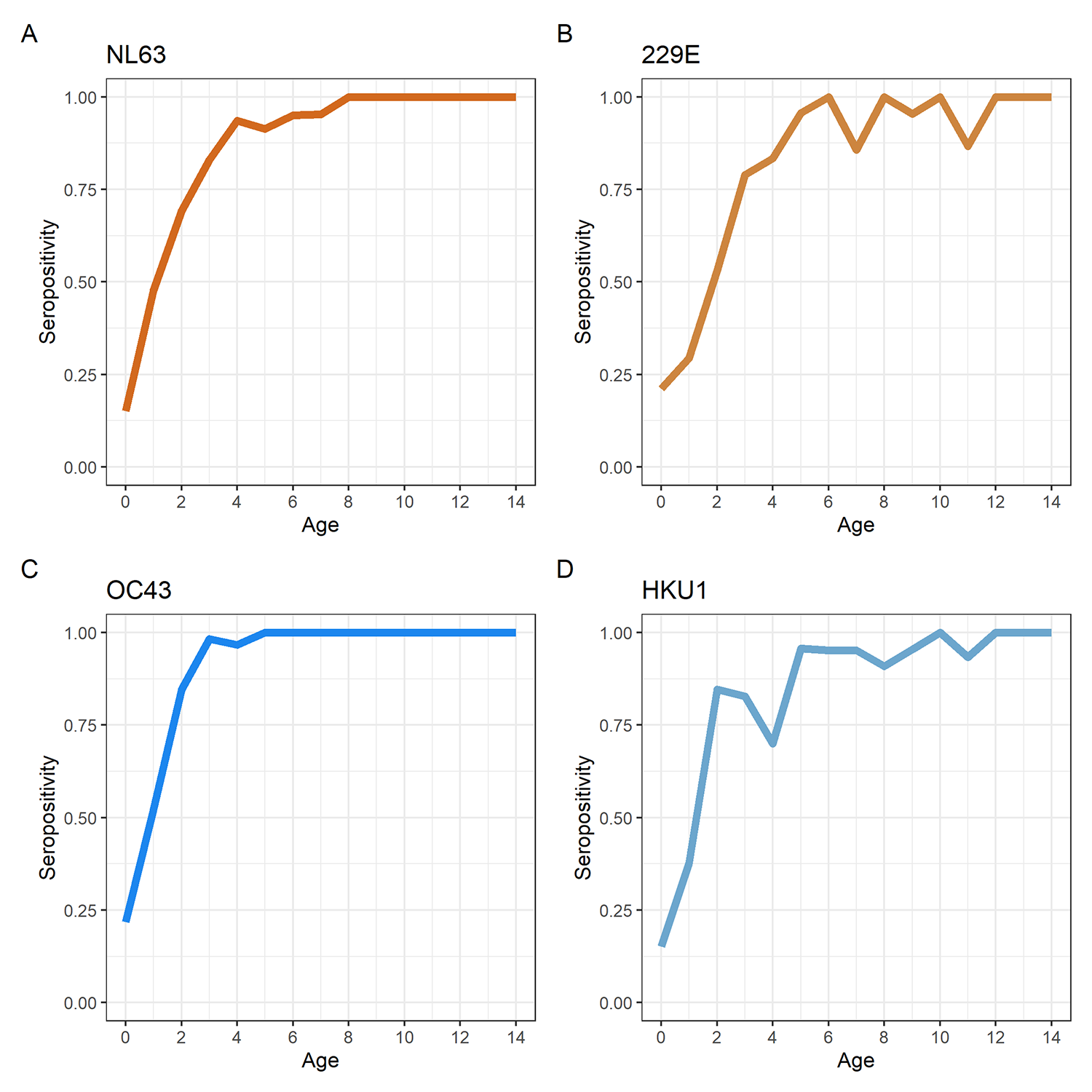

Supplement: Supplementary file 3 — Figure S3: ccCoV Seropositivity by Age, Type. Proportion of participants with ccCoV‐antibodies before ccCoV PCR + infection by one year age groups and type. A: NL63, B: 229E, C: OC43, D: HKU1 [file IRV-17-0-s004.tif]

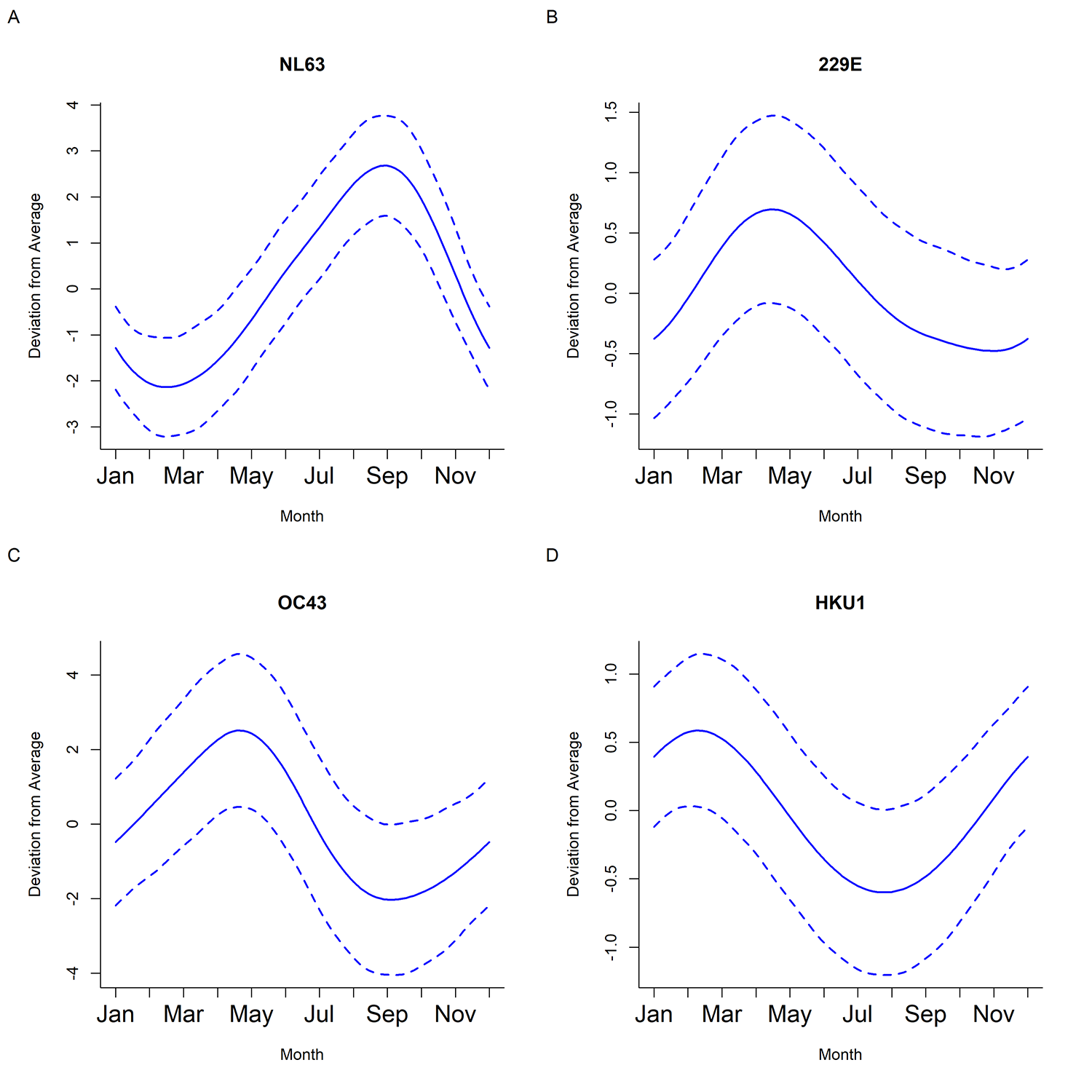

Supplement: Supplementary file 4 — Figure S4: Generalized Additive Model Analysis for Peak Month by ccCoV Type. Analysis uses month as the predictive variable for time series data for each ccCoV type. Dotted lines represent 95% confidence intervals. If confidence intervals at the peaks overlap with confidence intervals of the trough, there is no significant peak month. A: NL63, B: 229E, C: OC43, D: HKU1 [file IRV-17-0-s001.tif]

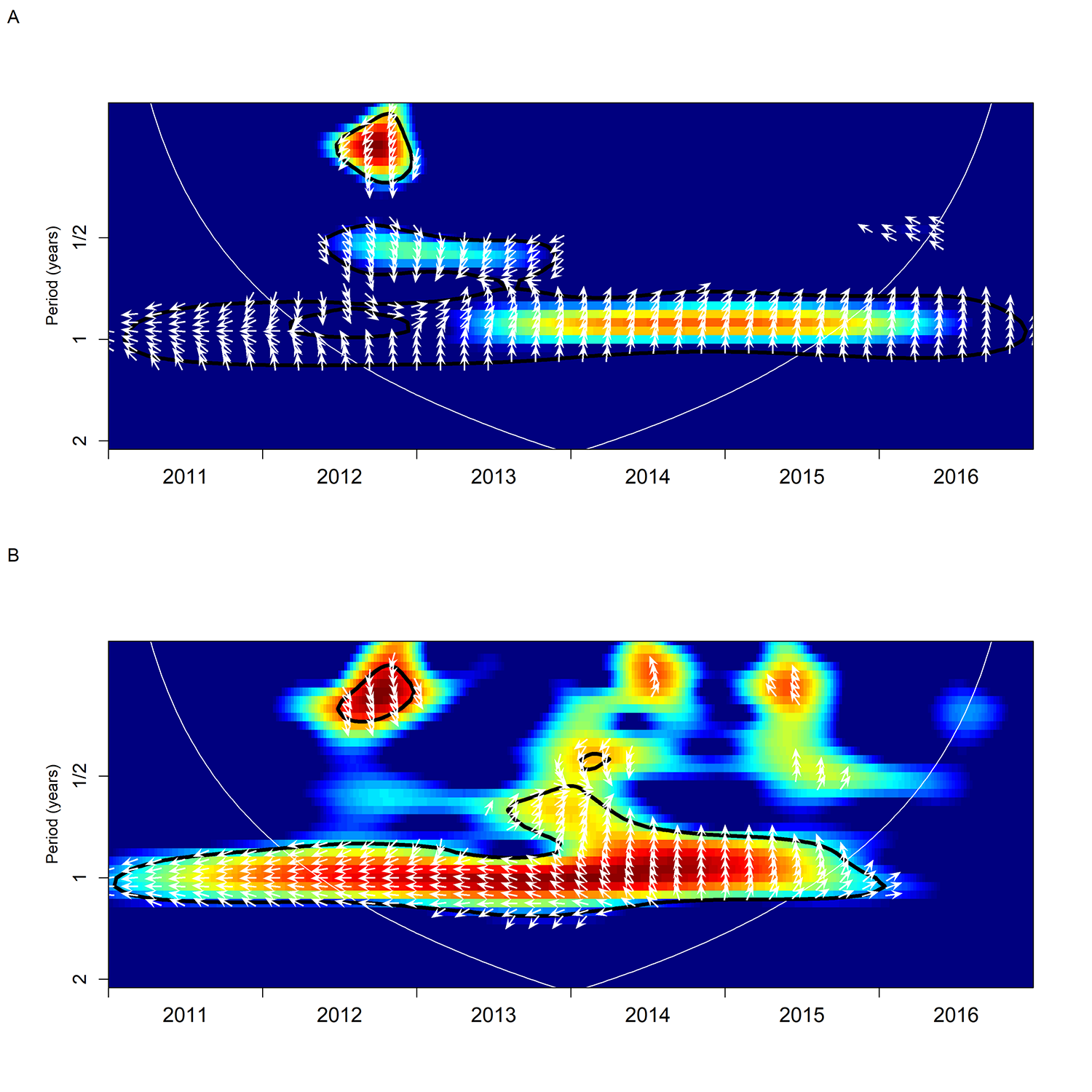

Supplement: Supplementary file 5 — Figure S5: Cross‐wavelet Analysis. Cross‐wavelet analysis of two ccCoV types to evaluate temporal relationship. White arrows pointing up at a period of 1 year represent a three‐month lag between the first list ccCoV type and the second. Arrows pointing to the left at a period of 1 year represent a six‐month lag between types. A: 229E‐NL63, B: OC43‐NL63 [file IRV-17-0-s005.tif]

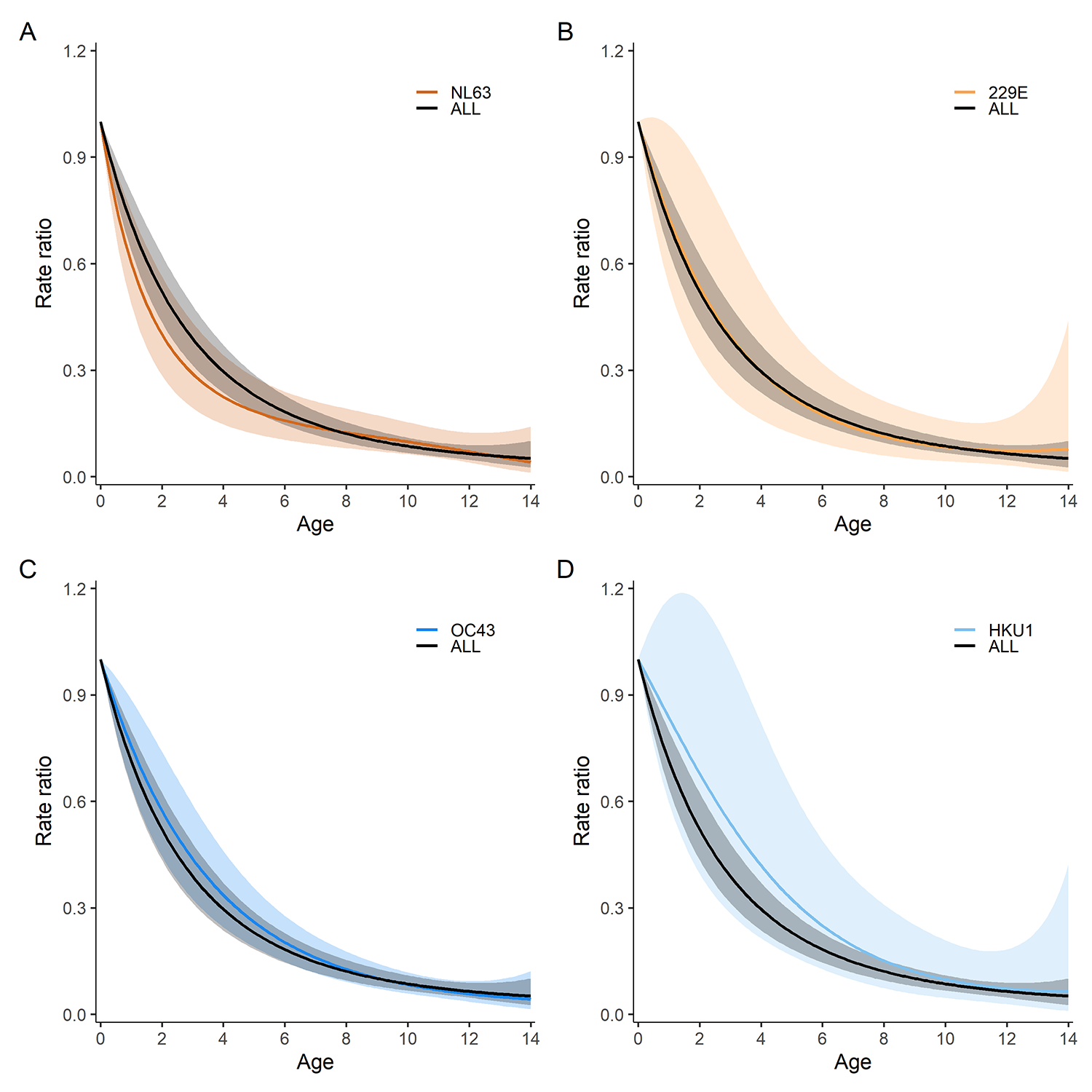

Supplement: Supplementary file 6 — Figure S6: Age‐period Incidence Model‐ Age Effects. Predicted rate ratios by age from age‐period model by ccCoV type. Black line represents predicted rate ratios for all ccCoV infections for comparison. A: NL63, B: 229E, C: OC43, D: HKU1 [file IRV-17-0-s008.tif]

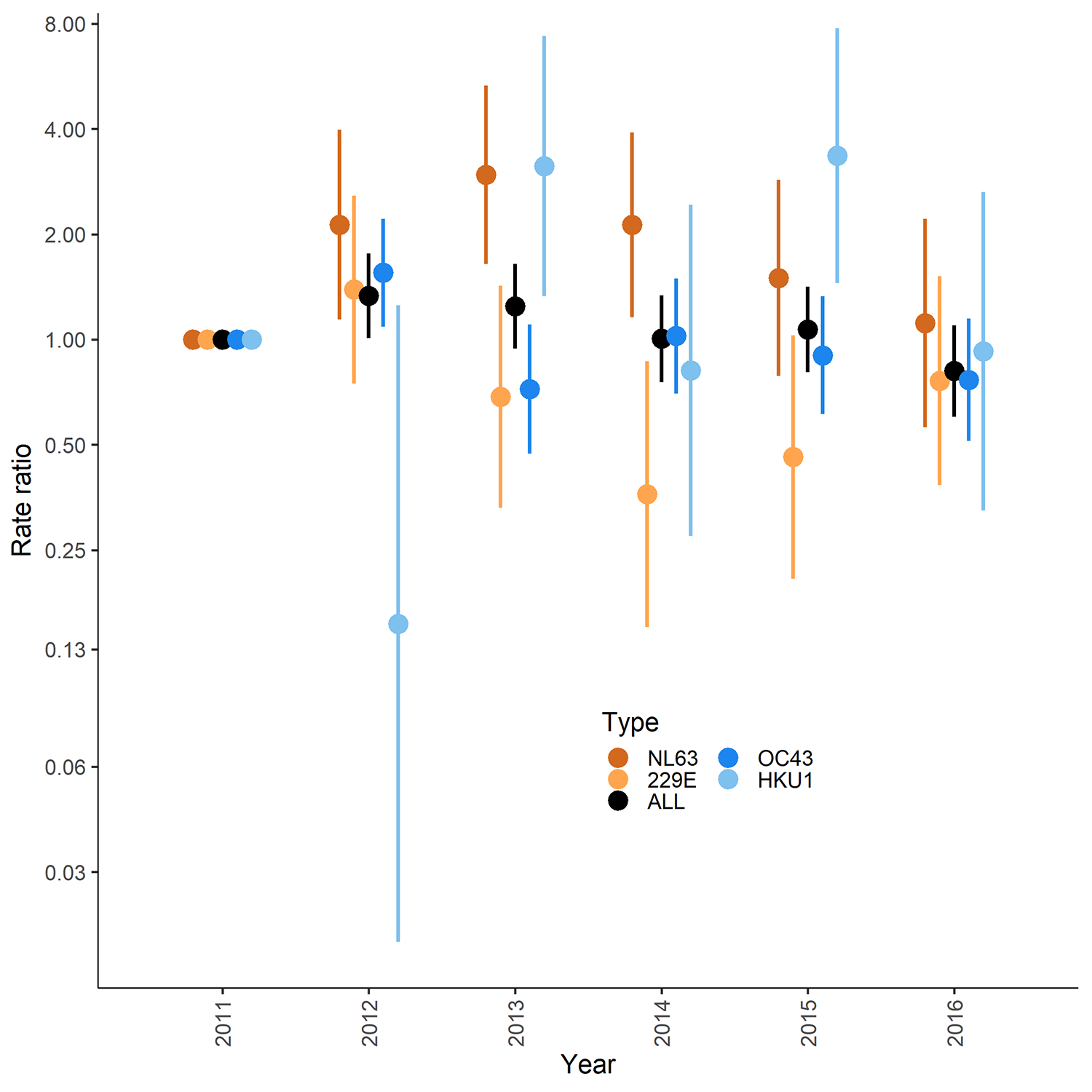

Supplement: Supplementary file 7 — Figure S7: Age‐period Incidence Model‐ Period Effects. Predicted rate ratios by year from age‐period model by ccCoV type. 2011 is the reference category. Black points and confidence intervals represent predicted rate ratios for all ccCoV infections for comparison. [file IRV-17-0-s006.tif]
