# Supplementary material for: Burden and seasonality of primary and secondary symptomatic common cold coronavirus infections in Nicaraguan children
Source: Influenza Other Respir Viruses. 2022 Dec 9;17(1):e13078. doi: 10.1111/irv.13086 (PMC9835451; doi:10.1111/irv.13086)
Supplement: Supplementary file 8 — Table S1. Incidence Rates by Sex [file IRV-17-0-s003.docx]

**Table S1**. Incidence Rates by Sex

|  | **Symptomatic ccCoV Incidence Rate per 1, 000 Person Years (95% CI)** | | | | |
| --- | --- | --- | --- | --- | --- |
|  | **All** | **NL63** | **229E** | **OC43** | **HKU1** |
| **Overall** | 61.1 (56.3, 66.2) | 16.8 (14.4, 19.5) | 8.6 (7.0, 10.7) | 32.0 (28.6, 35.8) | 6.9 (5.4, 8.7) |
| **Sex** |  |  |  |  |  |
| Female | 63.4 (56.8, 70.9) | 18.6 (15.2, 22.9) | 7.8 (5.6, 10.7) | 32.0 (27.3, 37.4) | 7.0 (5.0, 9.7) |
| Male | 58.6 (52.2, 65.9) | 14.9 (11.8, 18.7) | 9.5 (7.1, 12.7) | 32.1 (27.4, 37.5) | 6.8 (4.8, 9.6) |
|  | **ccCoV-Associated LRI Incidence Rate per 1, 000 Person Years (95% CI)** | | | | |
|  | **All** | **NL63** | **229E** | **OC43** | **HKU1** |
| **Overall** | 11.0 (9.1, 13.3) | 2.8 (1.9, 4.0) | 2.0 (1.2, 3.1) | 5.2 (4.0, 6.9) | 1.5 (0.9, 2.6) |
| **Sex** |  |  |  |  |  |
| Female | 9.9 (7.4, 13.1) | 2.0 (1.1, 3.8) | 1.2 (0.6, 2.7) | 4.5 (3.0, 6.8) | 2.0 (1.1, 3.8) |
| Male | 12.2 (9.5, 15.8) | 3.5 (2.2, 5.6) | 2.7 (1.6, 4.6) | 6.0 (4.2, 8.6) | 1.0 (0.4, 2.5) |
